# Integrating finite element analysis and physics-informed neural networks for biomechanical modeling of the human lumbar spine

**DOI:** 10.1016/j.xnsj.2025.100598

**Published:** 2025-02-17

**Authors:** Mohsen Ahmadi, Debojit Biswas, Rudy Paul, Maohua Lin, Yufei Tang, Talha S. Cheema, Erik D. Engeberg, Javad Hashemi, Frank D. Vrionis

**Affiliations:** a Department of Electrical and Computer Science, Florida Atlantic University, Boca Raton, FL, United States; b Department of Biomedical Engineering, Florida Atlantic University, Boca Raton, FL, United States; c Department of Ocean and Mechanical Engineering, Florida Atlantic University, Boca Raton, FL, United States; d Center for Complex Systems and Brain Sciences, Florida Atlantic University, Boca Raton, FL, United States; e Department of Neurosurgery, Marcus Neuroscience Institute, Boca Raton Regional Hospital, Boca Raton, FL, United States

**Keywords:** Lumbar spine, Finite element analysis, Physics-informed neural networks, Material properties, Spinal simulation, Vertebrae segmentation

## Abstract

**Background:**

Comprehending the biomechanical characteristics of the human lumbar spine is crucial for managing and preventing spinal disorders. Precise material properties derived from patient-specific CT scans are essential for simulations to accurately mimic real-life scenarios, which is invaluable in creating effective surgical plans. The integration of Finite Element Analysis (FEA) with Physics-Informed Neural Networks (PINNs) offers significant clinical benefits by automating lumbar spine segmentation and meshing.

**Methods:**

We developed a FEA model of the lumbar spine incorporating detailed anatomical and material properties derived from high-quality CT and MRI scans. The model includes vertebrae and intervertebral discs, segmented and meshed using advanced imaging and computational techniques. PINNs were implemented to integrate physical laws directly into the neural network training process, ensuring that the predictions of material properties adhered to the governing equations of mechanics.

**Results:**

The model achieved an accuracy of 94.30% in predicting material properties such as Young's modulus (14.88 GPa for cortical bone and 1.23 MPa for intervertebral discs), Poisson's ratio (0.25 and 0.47, respectively), bulk modulus (9.87 GPa and 6.56 MPa, respectively), and shear modulus (5.96 GPa and 0.42 MPa, respectively). We developed a lumbar spine FEA model using anatomical and material properties from CT and MRI scans. Vertebrae and discs were segmented and meshed with advanced imaging techniques, while PINNs ensured material predictions followed mechanical laws.

**Conclusions:**

The integration of FEA and PINNs allows for accurate, automated prediction of material properties and mechanical behaviors of the lumbar spine, significantly reducing manual input and enhancing reliability. This approach ensures dependable biomechanical simulations and supports the development of personalized treatment plans and surgical strategies, ultimately improving clinical outcomes for spinal disorders. This method improves surgical planning and outcomes, contributing to better patient care and recovery in spinal disorders.

## Introduction

Understanding the biomechanical behavior of the human lumbar spine is essential for diagnosing, treating, and preventing various spinal disorders. Accurate prediction of material properties from CT scans is crucial to ensure simulations reflect real-world conditions. Traditionally, Finite Element Analysis (FEA) has been a powerful tool for studying the mechanical properties and behavior of the lumbar spine [[1], [2], [3], [4], [5], [6]] and the cervical spine [3,[7], [8], [9]]. FEA has also been employed to investigate the biomechanical properties of modified cortical bone trajectory techniques [[10], [11], [12], [13]] in transforaminal lumbar interbody fusion, with findings indicating that these [3] techniques exhibit lower stress concentrations at adjacent segments compared to traditional methods [[14], [15], [16]]. However, the precision of these simulations heavily relies on the accuracy of the material properties used. Factors such as age, gender, and diseases like osteoporosis significantly influence bone morphology and mechanical properties [17]. For instance, Quantitative computed tomography (QCT) on clinical CT scanners can determine bone mineral density and some morphological properties of cancellous bone. However, the spatial resolution only reveals trabecular thickness and separation, not the detailed shapes of individual trabeculae seen in micro-CT or micro-MRI studies [18]. The challenge lies in obtaining precise measurements of spine material properties from clinical imaging, which often has limited spatial resolution. Addressing these variations enhances the predictive accuracy of biomechanical simulations, supporting better clinical outcomes.

Recent advancements in computational techniques have introduced Physics-Informed Neural Networks (PINNs) to enhance the accuracy of biomechanical models. PINNs integrate physical laws directly into the neural network training process, ensuring predictions adhere to the governing equations of mechanics. This approach addresses the limitations in traditional FEA models that often rely on assumed or uniform material properties, which do not account for patient-specific variations. Several studies have explored combining FEA and neural networks to improve modeling of material properties in biomedical applications. For instance, PINNs have been used in ultrasound elastography to predict the Young's modulus of tumors and in linear elasticity problems to discover spatial distributions of material properties [19]. Other researchers have employed deep learning methods to predict local strain fields and improve elasticity imaging accuracy [20]. Kamali and Laksari [21] further improved this approach by combining PINNs with UNet to propose an elasticity inversion neural network model, achieving faster and more accurate elasticity predictions. Other researchers have employed deep learning methods to address similar challenges.

Patel et al. [22] circumvented the solution of inverse problems in mechanics by using deep learning to predict local strain fields in elastic composite materials. Mohammadi et al. [23] used generative adversarial networks (GANs) for regularization in ultrasound elasticity imaging, improving the accuracy of elasticity reconstructions. Sneider et al. [24] applied convolutional neural networks (CNNs) to identify stiffness markers in breast cancer, demonstrating the potential of neural networks in biomedical elasticity imaging. Despite these advancements, applying FEA combined with neural networks to predict material properties of the human lumbar spine remains underexplored. Existing methods struggle with irregularly structured tissues and fail to incorporate patient-specific data, leading to less accurate simulations. Current lumbar spine FEA modeling is highly manual and labor-intensive, requiring the addition of ligaments, setting different contacts, and incorporating endplates and discs individually. This process is time-consuming and complex, especially for multiple patients, and there is no automated approach to streamline it.

In this paper, we propose a novel approach that combines FEA with PINNs to predict the material properties of the human lumbar spine more accurately. By leveraging detailed anatomical and material data from high-quality CT and MRI scans, this method enhances the fidelity of biomechanical simulations. Additionally, we introduce an automated coding approach that eliminates the need for manual work, simplifying the model while maintaining high accuracy. By utilizing patient-specific CT data, our method provides precise material properties, segmentation, stress fields, and surgical plans without invasive procedures, supporting more accurate and reliable biomechanical models.

## Methods

**Fig. 1:**
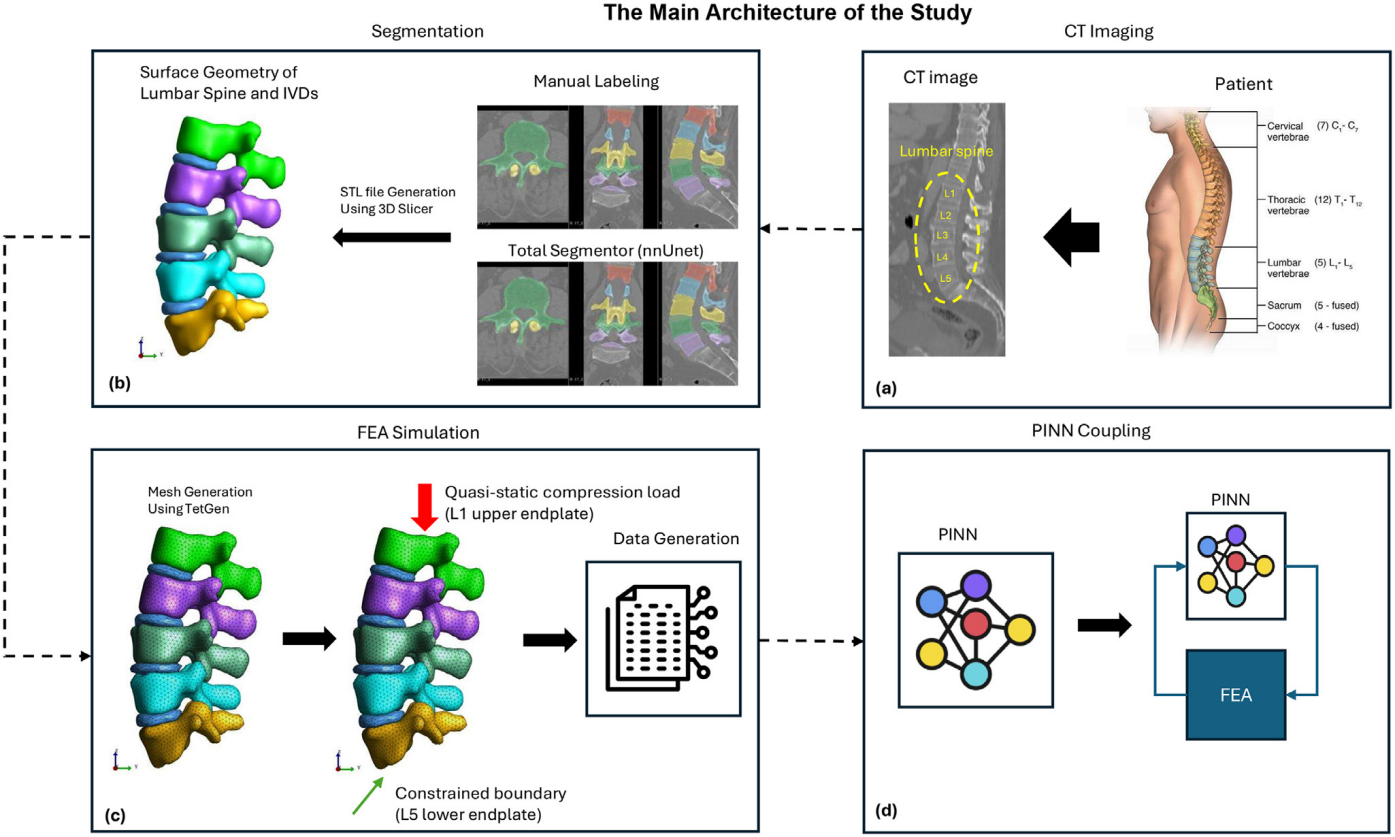
The main architecture of presented study including: (A) CT imaging; (B) Segmentation; (C) FEA simulation and (D) Integration of PINN and FEA.

In this study, we propose an integrated approach to model and analyze the lumbar spine using a combination of segmentation, FEA, and PINNs. The process begins with obtaining CT images of the lumbar spine (Fig. 1A). These methods are used for segmentation, where the surface geometry of the lumbar spine and intervertebral discs (IVDs) is extracted. This segmentation is performed manually and through an automated process using TotalSegmentator based on nnU-Net architecture (Fig. 1B). The segmented models are then converted into STL files using 3D Slicer. Subsequently, the segmented geometry undergoes mesh generation using TetGen, which prepares the model for FEA simulation (Fig. 1C). In the FEA simulation, external loads and constraints are applied to generate data that reflects the biomechanical behavior of the lumbar spine under various conditions. Then, we employ PINNs to integrate the simulation data with neural networks (Fig. 1D).

### Segmentation of lumbar spine

This model is based on the nnU-Net architecture, a robust and flexible framework designed for biomedical image segmentation tasks [25,26]. The nnU-Net automatically adapts to the specific requirements of the segmentation task, including the input image properties and the target structures [27]. The pretrained model used to segment the IVDs from the 3D images. We use pretrained models to extract IVDs in the test process from the CT images as shown in Fig. 1. After the initial segmentation steps, the segmented vertebrae and IVDs are processed separately to generate individual 3D models. Each vertebra and IVD are saved as an STL file, a widely used file format for representing 3D geometries. Modeling cartilage in lumbar spine simulations poses challenges due to the limited resolution of CT and MRI scans, which are insufficient for capturing the thin and complex geometry of cartilage layers. Additionally, cartilage contributes minimally to load transmission during spinal compression, with the primary mechanical response dominated by intervertebral discs and vertebral endplates. Studies have demonstrated that cartilage properties exert negligible influence on compressive load distribution [28].

### Mesh generation

This phase involves converting the segmented images into a finite element mesh. For this study, we will use TetGen [29] software to create tetrahedral elements, ensuring a detailed representation of the spinal geometry. Regarding Fig. 1, the lumbar spine is extracted from the CT image and meshed using tetrahedral cells. The complete vertebrae with surface mesh from 3 different views are given in Fig. 1A. The vertebral bodies of L1, L2, L3, L4, and L5 are created, including both anterior and posterior parts. Each vertebra had around 6,000–7,000 surface elements. For each IVD, around 2,000–3,000 elements were included. To apply tie contact between the bone and IVDs, the upper endplate and lower endplate of each vertebra were extracted after segmentation.

In this study, the facet joints were modeled using tied contact boundary conditions to simplify the computational complexity while maintaining the structural integrity of the lumbar spine model. This approach allowed for the omission of complex contact interactions, focusing primarily on axial compressive loading scenarios. The use of tied contacts ensures that relative movement between the facet joint surfaces is restricted, which is a reasonable approximation given the study's objectives. A surface shell was used for the vertebra because Young's modulus of the cortical layer is significantly higher than that of the cancellous bone. As a result, the forces applied to the cortical layer do not affect the interior of the vertebra, justifying the use of a shell for the vertebra. For the IVDs, an internal mesh was used, ensuring the internal part is meshed. The cartilage was not included in the simulation because sensitivity analysis indicated that the material properties of the articular cartilage do not significantly affect the force transmitted through the joint [29]. While forces were applied in the z-direction, ligaments were not involved in the motion. Ligaments are primarily tension-based structures, meaning they resist tensile forces and provide stability by preventing excessive movement [30]. However, in this simulation, their exclusion was based on the focus being on compressive forces and the mechanical behavior of the vertebrae and IVDs.

#### Annotated the upper and lower endplate for vertebra

**Fig. 2:**
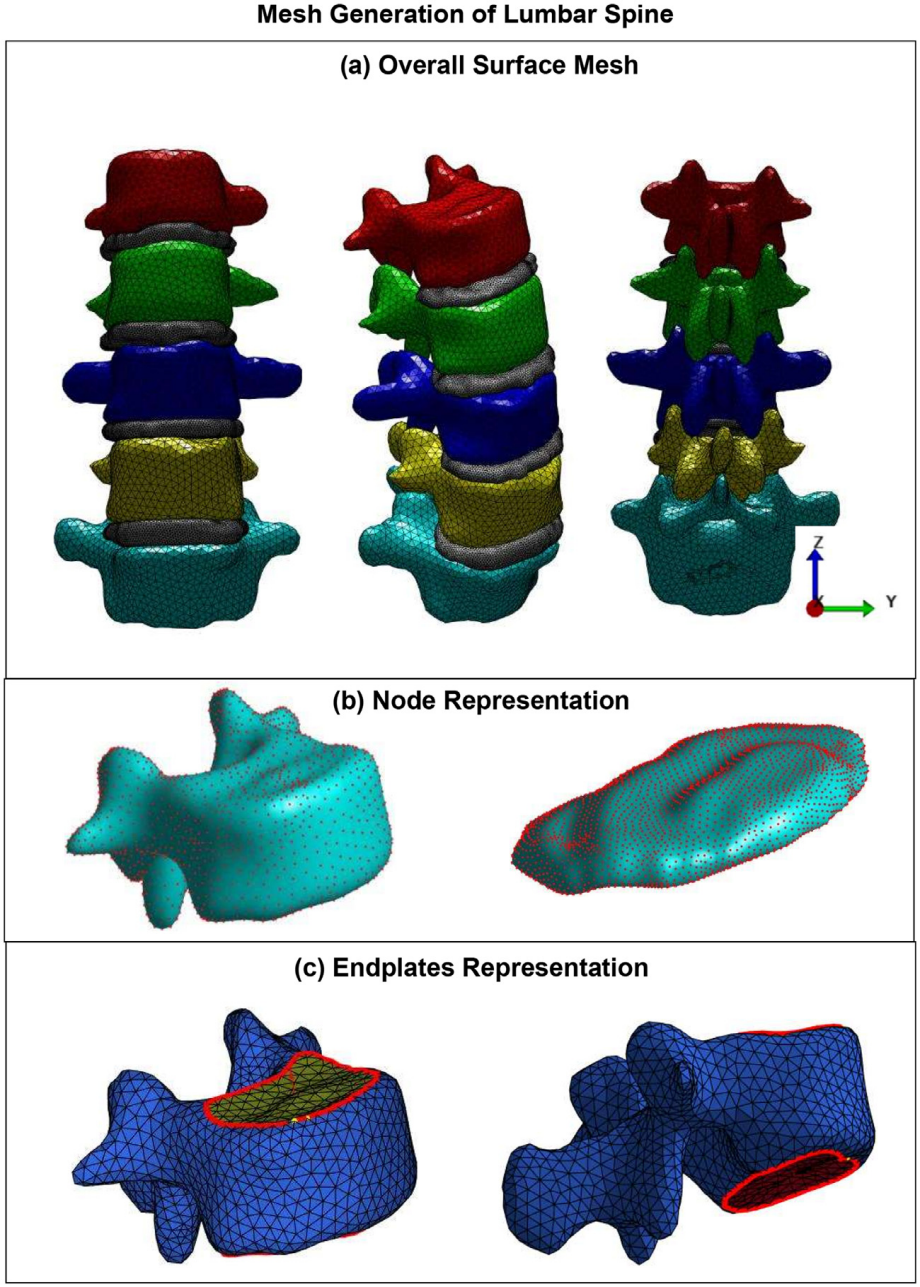
Mesh generation (A) Overall surface mesh, (B) The nodes and surface of the meshes, (C) Annotation of the upper and lower endplate.

To approximate the upper and lower endplates of vertebrae where IVDs attach, a hardcoded method is employed. This method involves detecting surface elements based on their angular orientation relative to the z-axis and their distance from the origin. Regarding Fig. 2C, for the identification of the upper and lower endplates, elements that have normally formed a small angle with the z-axis and are located within a certain distance from the origin are identified as part of the top surface. Considering the images taken in [[30], [31], [32]] the nucleus and annulus of vertebrae could not be distinguished because the images were only taken in representative cross sections. Therefore, this model contains only 1 material for the bones and 1 material for the IVDs.

In the next step, we configured the FEBio software [33] by defining the material properties and boundary conditions of the lumbar spine geometry. Based on the Junior and Trajanovic [30], we defined the vertebrae as a linear elastic isotropic material with a specific Young's modulus and Poisson's ratio. This simplification allows for the evaluation of global load distribution and structural stiffness under axial compressive loading without the excessive computational cost associated with nonlinear models. Previous studies have demonstrated that assuming linear elasticity provides acceptable approximations for overall mechanical behavior, particularly when focusing on load-bearing capacity rather than failure prediction. The material properties used in the model were sourced from well-established reference literature, ensuring reliability and consistency across simulations. For the IVDs, we utilized the Ogden material model to capture their nonlinear elastic behavior [34]. Unlike the classical model where fibers are embedded in a ground substance, in the literature the annulus was modeled differently using the Ogden material model. In this paper we also use Ogden material for the IVDs. This model is based on the strain energy potential for distributed collagen fibers within the ground substance [35]. The key advantage of this approach, compared to phenomenological models, is that it attributes material properties directly to the fibers and matrix constituents. Regarding study of Baroud et al. [36] and Junior and Trajanovic [30], in this paper, we applied displacement in the load FE analysis of the L1-L5 segment by applying a quasi-static compression load in steps of 0.1 mm over 10-time steps. Fig. 2B illustrates the mesh vertices and faces of the vertebrae and IVDs.

In this study, the material properties of the lumbar spine components, including cortical and cancellous bone, were obtained from established reference literature. Computed Tomography (CT) values, expressed in Hounsfield Units (HU), are widely utilized in clinical and research applications to estimate bone mineral density (BMD) and mechanical properties such as Young's modulus and Poisson's ratio. The HU values represent the X -ray attenuation characteristics of tissues, with cortical bone typically ranging from +700 to +2000 HU and cancellous bone from +100 to +300 HU, which aligns with the values adopted in this study. According to Rho et al. [37], the correlation between HU values and mechanical properties differs for cortical and cancellous bone. Specifically, cortical bone exhibits a low correlation ($R^{2}<0.2$), suggesting that HU values alone are not sufficient for accurately predicting properties such as Young's modulus. Conversely, cancellous bone demonstrates a stronger correlation ($R^{2}>0.6$), making HU values a more reliable predictor for its mechanical properties. The mechanical properties of cancellous bone can be estimated using empirical relationships such as Young's modulus $\left(\text{MPa}\right)=a\cdot HU^{b}$ and density $(\text{kg}/m^{3})=c+d\cdot \text{HU}$.

## Results

### Material property estimation using neural networks

The purpose of this step is to analyze the mechanical behavior of the spine under different loading conditions. A robust dataset for training neural networks can be generated by simulating different loading scenarios and recording the displacements, stresses, and strains that result from them. Using these networks, we will be able to predict material properties such as Young's modulus, Poisson's ratio, bulk modulus, and shear modulus. Cortical vertebral shells are commonly modeled as linear elastic isotropic or transversely orthotropic materials. A vertebra's cortical bone is characterized by superior strength and stiffness. Young's modulus (E) and Poisson's ratio (ν) are the primary parameters used to describe its mechanical properties.

**Fig. 3:**
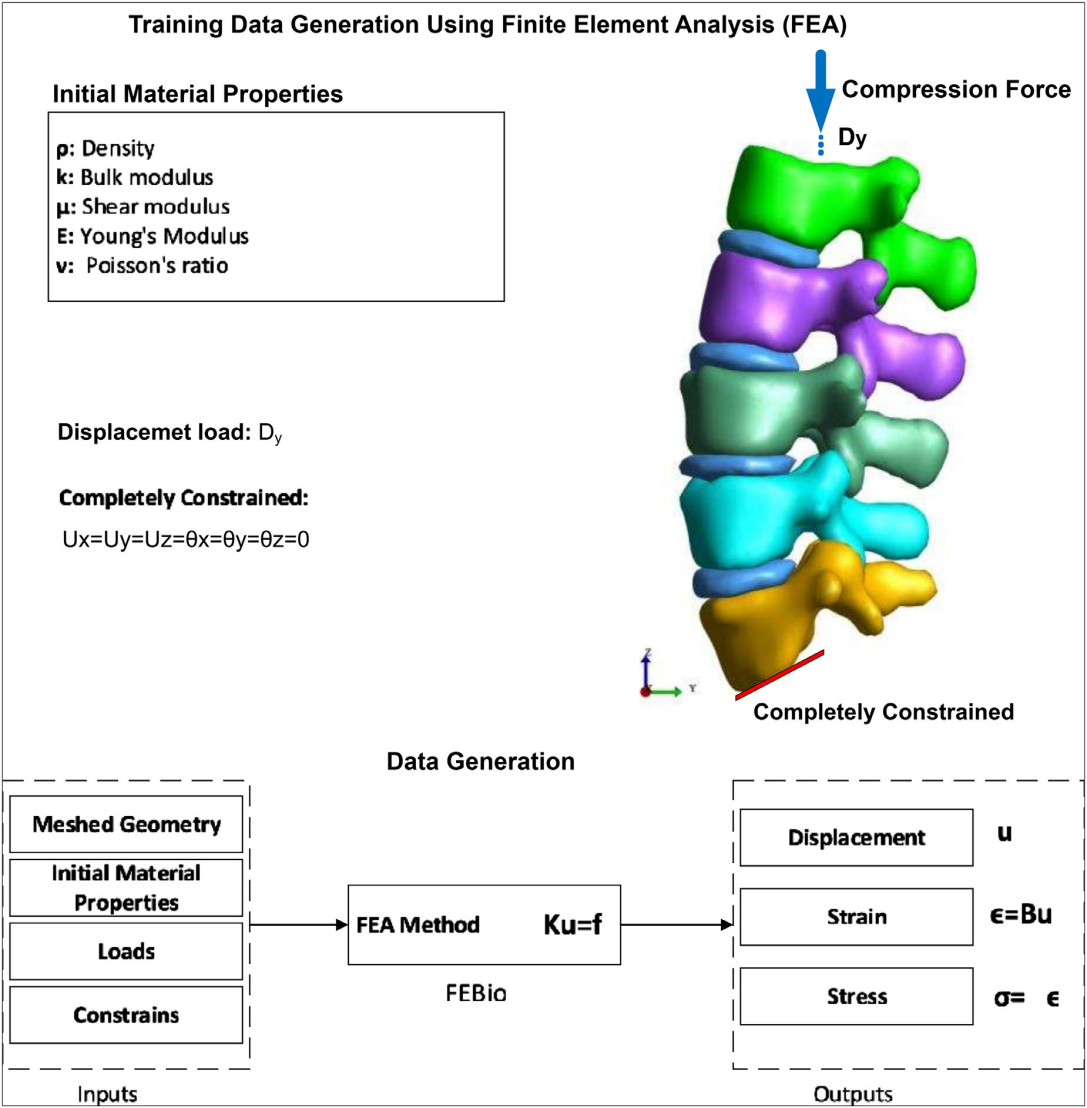
The process of training data generation based on FEA.

To generate the training data, we simulate different loading values on synthetic spine models using the FEA. This involves creating a detailed meshed geometry of the spine and applying various initial material properties and boundary conditions. The simulation aims to replicate realistic physiological loading scenarios to capture the mechanical response of the spine. The process of training data generation based on FEA is illustrated in Fig. 3. The loading conditions can be applied to the spine model include nodal forces ($F_{x},\;F_{y},\;F_{z}$), flexion/extension moments ($M_{x}$), lateral bending moments ($M_{z}$), axial rotation moments ($M_{y}$), follower loads (P), surface forces (T), and body forces ($b_{x},\;b_{y},\;b_{z}$) like gravitational acceleration. Additionally, specific regions of the model are completely constrained to simulate real-world boundary conditions. Loads are generally applied to the upper endplate of the highest vertebrae of interest. Moreover, the lower endplate is used as constrained boundary so that the displacement for all direction ($u_{x}=u_{y}=u_{z}=0$) and rotational degrees of freedom ($\theta_{x}=\theta_{y}=\theta_{z}=0$) in all directions are constrained. In this study, the lower endplate of the L5 vertebra was fully constrained to simulate the physiological support provided by the sacrum. This constraint was applied to restrict all translational and rotational degrees of freedom, ensuring structural stability and realistic load transfer within the lumbar spine model. The full constraint at the L5 lower endplate is a widely adopted approach in finite element modeling of the spine, as it replicates in vivo boundary conditions with high accuracy.

**Fig. 4:**
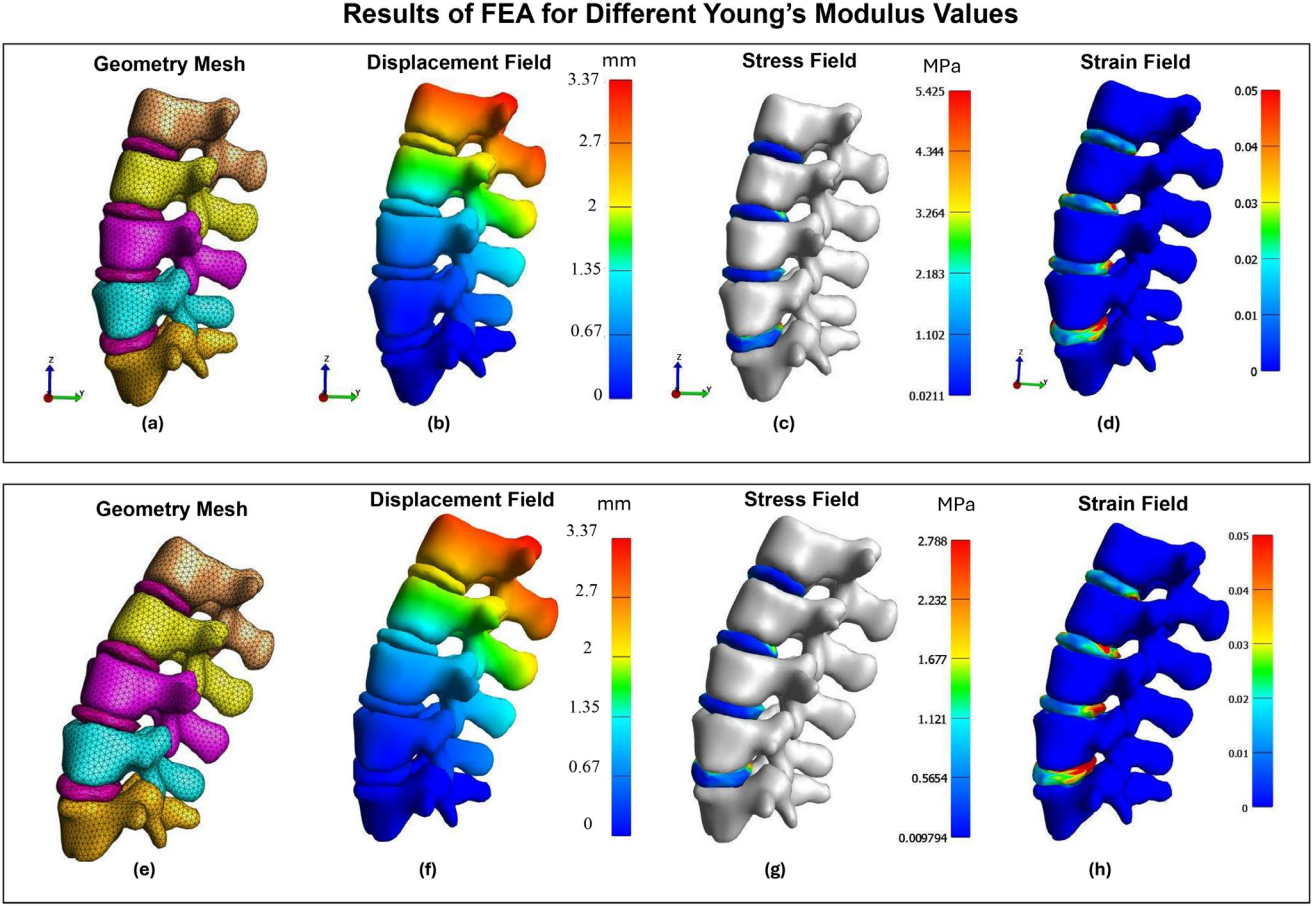
The results of FEA for 2 cases with high(top) and low(bottom) young module: A,E) Geometry mesh; B,F) Displacement magnitude; C,G) Stress magnitude; D,H) Strain magnitude.

This assumption allows for the effective evaluation of load distribution and mechanical responses under various loading scenarios while maintaining computational efficiency. Also, the material properties used were within the range based on Junior & Trajanovic [30]. For instance, the Young's modulus for bone was set between 10 and 25 GPa. For the disc, the range was between 1 and 4.1 MPa. The Poisson's ratio ranged from 0.1 to 0.4 for bone and from 0.45 to 0.48 for the IVDs. Fig. 4A–D illustrate the FEA results of the lumbar spine, depicting displacement, stress, and strain magnitudes. The displacement magnitude (b) shows how much each part of the spine moves under a given load, with higher displacements indicated by warmer colors. The stress magnitude (c) visualizes the distribution of stress within the lumbar spine, where regions experiencing higher stress are highlighted in red. The strain magnitude (d) indicates the deformation per unit length, with higher strains shown in warmer colors. Regarding these results, while applying force on the L1 and fixing the L5, the maximum displacement is recorded for the L1. Moreover, most of the stress values can be seen in the discs. Correspondingly, the increased stress causes more strain in the discs, as shown in Fig. 4D.

### Neural network design and training

With the training data generated from the FEA simulations, we design and train a neural network to estimate the material properties based on the recorded mechanical responses. The input data for the neural network consists of the mechanical responses (displacements, strains, and stresses) from the FEA simulations. The output data includes the corresponding material properties (Young's modulus, Poisson's ratio, bulk modulus, and shear modulus). The neural network, represented as *y* = N(x;θ), where *y* is the output vector of material properties, *x* is the input vector of mechanical responses, and θ represents the network parameters, is trained to learn the mapping between the input and output data. In this research we use 16 input features including, applied load ($F_{ext}$), displacement ($u_{x},\;u_{y},\;u_{z}$), normal stresses ($\sigma_{x},\;\sigma_{y},\;\sigma_{z}$), shear stresses ($\sigma_{xy},\;\sigma_{yx},\;\sigma_{xz}$), normal strains (ε*_x_*, ε*_y_*, ε*_z_*), shear strains (ε*_xy_*, ε*_yx_*, ε*_xz_*).

The model predicts the material properties (Young's modulus, Poisson's ratio, bulk modulus, shear modulus and density). Therefore, we use 10 output features including, Young's modulus of bone ($E_{bone}$), Young's modulus of disc ($E_{disc}$), Poisson ratio of bone ($\nu_{bone}$), Poisson ratio of disc ($\nu_{disc}$), bulk modulus of bone ($k_{bone}$), bulk modulus of discs ($k_{disc}$), shear modulus of bone ($\mu_{bone}$), shear modulus of discs ($\mu_{disc}$), density of bone($\rho_{bone}$), and density of disc ($\rho_{disc}$). Multiple layers with nonlinear activation functions to capture complex relationships between input and output. In this paper we have trained different number of hidden layers and neurons to find the optimum model for 2 images of a patient. To create a dataset to train a neural network, we need to define different load and material properties. Therefore, we applied quasi-static compression loads of 0.2, 0.4, 0.6, 0.8, 1.0, 1.2, 1.4, 1.6, 1.8, 2.0, 2.2, 2.4, 2.6, and 2.8 mm, each applied in 10-time steps, and finally, the displacement, stress, and strain were recorded for each condition.

### Physics-informed neural networks

To ensure that the neural network predictions adhere to physical laws, we integrate the governing equations of mechanics into the training process. This approach, known as PINN, incorporates physical constraints directly into the loss function, ensuring that the network outputs are consistent with the underlying mechanics. The PINN loss function is then modified to include these physical constraints:

(1) $$\begin{array}{ccc} \text{Loss}\left(\theta \right) & = & \frac{1}{N}\sum_{i=1}^{N}{\left|N\left(x_{i};\theta \right)-\left[E_{i},\nu_{i},k_{i},\mu_{i}\right]\right|}^{2}\\ & & +\;\lambda_{1}\left|k_{i}-\frac{E_{i}}{3\left(1-2\nu_{i}\right)}\right|+\lambda_{2}\left|\mu_{i}-\frac{E_{i}}{2\left(1+\nu_{i}\right)}\right| \end{array}$$

where $\lambda_{1}$ and $\lambda_{2}$ is a regularization parameter that controls the contribution of the constraints to the overall loss. By integrating these physical laws into the neural network training, we ensure that the network not only learns the mapping between mechanical responses and material properties but also adheres to the fundamental principles of mechanics.

**Fig. 5:**
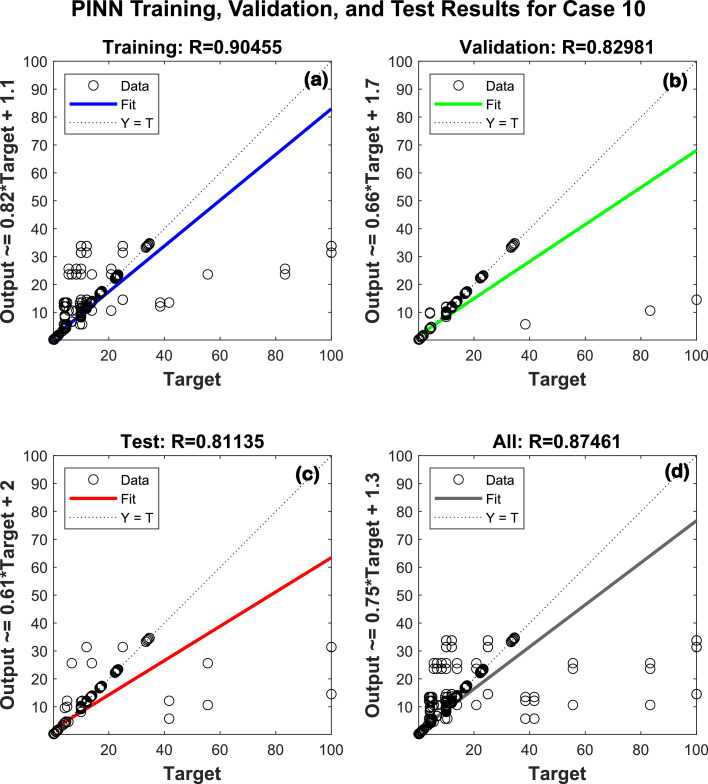
PINN training, validation and test results for Case 10 with 3 hidden layers of [64 32,16] neurons.

Table 1 presents the results of training using PINN across various hidden layer architectures. Each case involves 404 analyses with 16 input features and 10 output features for 2 images of a patient. Table 1 shows the training, validation, and test accuracies for different hidden layer configurations. The architecture (64-32-16) (Case 10 in Fig. 5) achieved the highest performance, with training accuracy of 90.46%, validation accuracy of 82.98%, and test accuracy of 81.14%.

**Table 1:** Result of training using artificial neural network

Cases	Number of analyses	Number of input features	Hidden layers architecture	Number of output features	Train accuracy	Validation accuracy	Test accuracy
1	404	16	[16]	10	85.92%	97.89%	84.85%
2	404	16	[32]	10	80.94%	95.93%	82.93%
3	404	16	[64]	10	88.33%	86.94%	88.92%
4	404	16	[16-8]	10	87.13%	90.77%	86.03%
5	404	16	[16-16]	10	88.40%	96.43%	77.64%
6	404	16	[32-16]	10	87.54%	96.79%	84.81%
7	404	16	[32-32]	10	90.01%	81.18%	82.02%
8	404	16	[64-32]	10	89.40%	81.21%	82.46%
9	404	16	[64-64]	10	88.41%	95.37%	83.42%
10	404	16	[64-32-16]	10	90.46%	82.98%	81.14%
11	404	16	[128-64-32]	10	87.32%	97.00%	84.63%

**Fig. 6:**
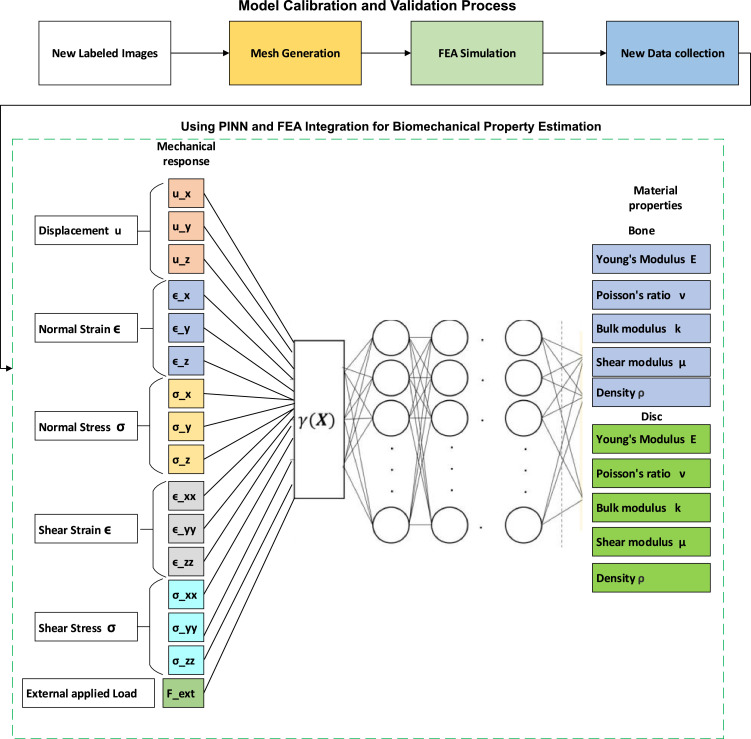
The process of the model calibration and validation.

The calibration dataset is prepared by running additional FEA simulations under various loading conditions or using experimental data collected from physical tests on spine samples regarding Fig. 6. Calibration involves fine-tuning the neural network and FEA parameters to ensure that the model accurately reflects the physical behavior of the spine.

**Fig. 7:**
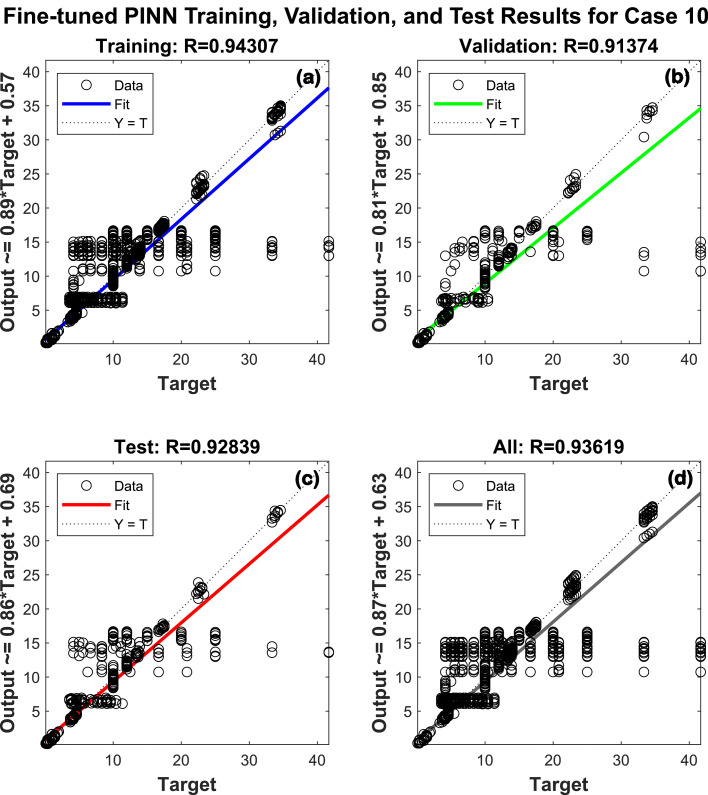
Fine-tuned PINN training, validation and test results for Case 10 with 3 hidden layers of [64 32,16] neurons.

This process is iterative, involving adjustments and re-training of the neural network based on the validation outcomes. Regarding Table 2 and Fig. 7, there are 678 analyses involved in each case, which include 16 input features and 10 output features. An overview of the training, validation, and test accuracies for various hidden layer configurations is presented in Table 2. With a training accuracy of 94.30%, a validation accuracy of 93.37%, and a test accuracy of 92.83%, Case 10 achieved the highest performance regarding Fig. 7.

**Table 2:** Result of training using artificial neural network after fine-tuning

Cases	Number of analyses	Number of input features	Hidden layers architecture	Number of output features	Train accuracy	Validation accuracy	Test accuracy
1	678	16	[16]	10	93.98%	94.43%	90.95%
2	678	16	[32]	10	93.65%	93.08%	92.74%
3	678	16	[64]	10	94.00%	94.10%	91.22%
4	678	16	[16-8]	10	93.85%	93.61%	92.75%
5	678	16	[16-16]	10	94.11%	92.27%	91.83%
6	678	16	[32-16]	10	94.16%	93.40%	91.77%
7	678	16	[32-32]	10	93.75%	94.01%	92.78%
8	678	16	[64-32]	10	93.71%	94.65%	91.36%
9	678	16	[64-64]	10	94.05%	92.34%	92.81%
**10**	**678**	**16**	[64-32-16]	**10**	**94.30%**	**93.37%**	**92.83%**
11	678	16	[128-64-32]	10	93.74%	94.06%	91.44%

**Fig. 8:**
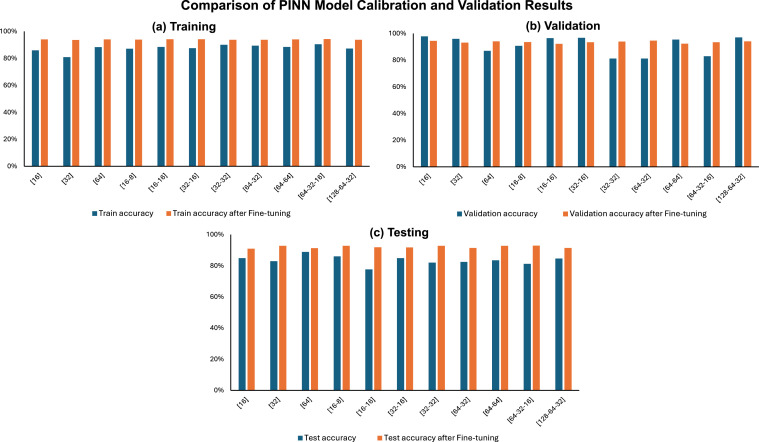
The integration of PINN and FEA.

The analysis of the neural network results before and after fine-tuning demonstrates improvements in performance across different architectures. Before fine-tuning, the training accuracies varied notably across the different architectures, ranging from 80.94% to 90.46% regarding Fig. 8. The validation accuracies also showed considerable variance, from 81.18% to 97.89%, and test accuracies ranged from 77.64% to 88.92%. On the other hand, the [64-32–16] architecture achieved the highest training accuracy of 90.46%, but its validation and test accuracies were 82.98% and 81.14%, respectively. After fine-tuning, there was an improvement in the performance metrics for almost all architectures. The training accuracies improved, with the highest being 94.30% for the [64-32–16] architecture. This configuration also demonstrated higher validation and test accuracies, 93.37% and 92.83%, respectively, making it the most effective architecture for predicting the biomechanical behavior of the lumbar spine.

### Integration of FEA and PINN

**Fig. 9:**
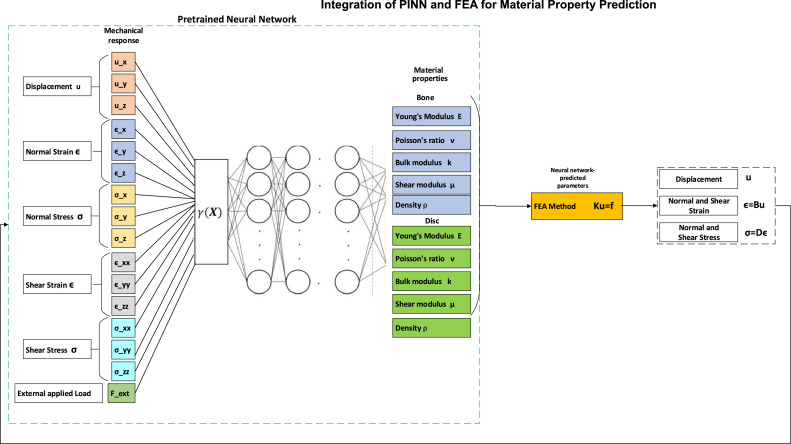
PINN model calibration and validation results.

This step in the process is the integration of the trained PINN with FEA to predict material properties and simulate the mechanical behavior of the spine. The integration process involves coupling the trained neural network with the FEA software, allowing the neural network to predict material properties based on the observed mechanical response during the simulation. Once the integration is set up, FEA simulations are run using fine-tuned PINN network. Regarding Fig. 9A, the simulation process begins with setting up and initializing the initial mechanical response data obtained from the FEA simulations using the initial material properties. This data is fed into the neural network to predict the updated material properties. The FEA solver runs simulations using these updated material properties provided by the neural network.

The mechanical response (displacements, strains, and stresses) is calculated for each loading condition. The mechanical response data from the FEA simulations is fed back into the neural network, which updates its predictions for the material properties based on the new data. This feedback loop continues, with the FEA simulations and neural network predictions iteratively refining each other. The iterative process continues until the simulation results converge to a stable solution that accurately reflects the physical behavior of the spine. The integration of FEA and PINNs employs an iterative feedback loop that effectively dampens numerical and modeling errors, ensuring convergence of material property predictions and biomechanical responses. This iterative process allows discrepancies in initial predictions to be minimized in subsequent iterations, leading to stable and accurate results. The iterative process can be described mathematically as:

(2) $$x_{n+1}=f^{-1}\left(f\left(x_{n}\right)\right),$$

where $x_{n}$ represents the predicted material properties at the n-th iteration, f represents the FEA simulation, and $f^{-1}$ denotes the PINN-based prediction of material properties from the simulated mechanical responses. Let $x^{*}$ denote the true material properties. Define the error at iteration n as:

(3) $$e_{n}=x_{n}-x^{*}.$$

Linearizing the iterative process, the error propagation can be expressed as:

(4) $$e_{n+1}=Ae_{n},$$

where A is the error propagation matrix, determined by the sensitivity of f and $f^{-1}$. For the iterative process to converge, the spectral radius of A must satisfy:

(5) ρ(A)<1.

This condition ensures that the error decreases with each iteration, leading to the convergence of $x_{n}$ to $x^{*}$.

**Fig. 10:**
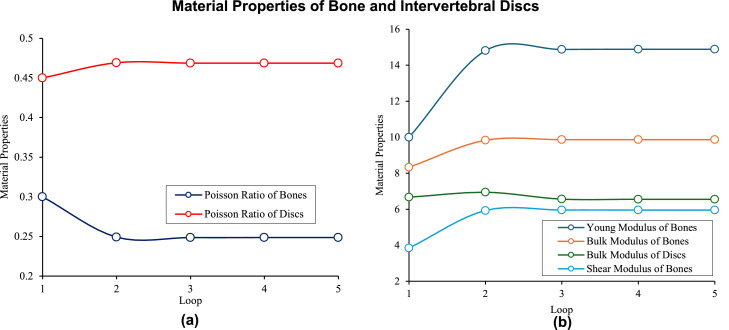
The material properties of bone and IVDs, young and bulk modulus, and Poisson ratios.

Figs. 10A and B show the optimum material properties that are calculated using integration of FEA and PINN. Regarding the results, after 3 loops of the hybrid system the material properties and correspondingly mechanical responses are converged to a constant value. Therefore, regarding the results using our presented integrated system we can estimate the material properties using the mechanical responses. The final value for the bone includes Young's modulus around 14.88 GPa, Poisson's ratio approximately 0.25, bulk modulus around 9.87 GPa, and shear modulus around 5.96 GPa. Moreover, for the IVDs, the final values are Young's modulus around 1.23 MPa, Poisson's ratio approximately 0.47, bulk modulus around 6.56 MPa, and shear modulus around 0.42 MPa. The convergence of these values demonstrates the effectiveness of the integrated FEA-PINN approach in accurately predicting the material properties, ensuring reliable biomechanical simulations of the lumbar spine.

## Discussion

This study presents a methodology that integrates Finite Element Analysis (FEA) with Physics-Informed Neural Networks (PINNs) to estimate the material properties of the human lumbar spine. By combining advanced computational techniques and patient-specific imaging data, this approach accurately predicts biomechanical behavior without requiring invasive procedures or extensive manual input. The proposed model achieves over 94% accuracy in predicting key material properties, including Young's modulus, Poisson's ratio, bulk modulus, and shear modulus. These predictions provide reliable parameters for spinal simulations, addressing the variability that traditionally characterizes validation results for spinal material properties.

PINNs enhance this framework by embedding physical laws directly into the neural network training process. This ensures that predicted material properties adhere to the governing equations of mechanics, eliminating discrepancies between simulated and physiological behavior. By deriving training data from an extensive review of literature-based reference values, this study maintains consistency and reliability across simulations, enabling a robust estimation of material properties.

The automated nature of this methodology represents a significant improvement over traditional lumbar spine FEA modeling, which required extensive manual effort, including the addition of ligaments, manual segmentation, and configuring contacts. These processes were labor-intensive and prone to variability. By automating segmentation, meshing, and the integration of endplates and intervertebral discs (IVDs), this study significantly reduces preparation time while maintaining computational efficiency. The use of nnU-Net-based segmentation ensures precise identification of spinal structures, while TetGen generates high-quality meshes for subsequent FEA simulations.

The study focuses on compressive forces due to their predominant role in spinal mechanics and the high accuracy achieved in predicting material properties. The simplification of facet joint boundary conditions using tied contact was adopted to enhance computational efficiency and focus on axial loading effects. While this assumption reduces the complexity of the model, it may limit the accurate representation of facet joint mechanics, such as shear forces and rotational movements While additional moments would require extensive data for training, the exclusion of these other forces and ligaments is justified for this study's scope. This simplification does not significantly affect the model's reliability in predicting compressive stress and strain distributions.

In modeling, cortical and cancellous bones are treated as a single material for FEA. This decision is based on the observation that when forces are applied to the outer layer, the inner layers do not experience significant forces, allowing the vertebral body to be effectively modeled as cortical bone. This aligns with previous studies using rigid body models for spine FEAs, showing minimal impact on simulation accuracy. For modeling the intervertebral discs (IVDs), the Ogden material model is used to capture their nonlinear elastic behavior, directly attributing material properties to the fibers and matrix constituents. This approach, supported by literature, provides a more accurate representation of the disc's mechanical behavior and justifies the exclusion of fibers by accounting for distributed collagen fibers within the ground substance. While the assumption of linear elastic isotropic properties offers computational efficiency and simplifies the finite element modeling process, it does not fully capture the anisotropic and nonlinear behavior of vertebral bone under complex loading conditions. Bone exhibits distinct mechanical responses depending on directionality and load magnitude, which cannot be entirely represented by a linear model. As a result, localized stress concentrations and failure mechanisms may not be accurately predicted. Future work will focus on implementing advanced material models, such as nonlinear and anisotropic constitutive equations, to enhance the model's predictive accuracy and provide a more realistic representation of vertebral biomechanics.

By integrating FEA with PINNs, the proposed framework enables iterative refinement of material property predictions based on observed displacements, stresses, and strains. This feedback loop ensures convergence to physiologically accurate values, with predictions closely matching reference data. For example, the model successfully predicts Young's modulus of 14.88 GPa for cortical bone and 1.23 MPa for IVDs, with corresponding Poisson's ratios of 0.25 and 0.47. These values align well with experimental measurements reported in the literature, reinforcing the validity of the methodology.

## Clinical relevance

The clinical implications of this research are substantial. The ability to derive patient-specific material properties directly from CT scans enables more precise diagnostic and treatment planning. For example, the accurate prediction of stress fields and biomechanical responses supports personalized surgical strategies, including optimized implant selection and tailored rehabilitation protocols. Additionally, the streamlined workflow reduces modeling time and variability, enhancing its applicability in clinical settings where timely decision-making is critical. This study bridges the gap between traditional FEA and machine learning-based approaches, offering a hybrid framework that improves both accuracy and efficiency. By leveraging the complementary strengths of physics-based simulations and data-driven predictions, this methodology establishes a foundation for advancing spinal biomechanics research and clinical applications. Future work will focus on optimizing implants based on varying material properties identified in different patients, further personalizing patient care. The potential to integrate additional loading conditions and diverse patient data in future models will enhance their robustness and applicability, improving clinical decision-making and patient outcomes. This approach supports a shift towards more personalized and precise spine care, ultimately improving the quality of life for patients with spinal disorders.

While this study presents significant advancements, it also has limitations. The current model does not use deformation data from CT images, which limits the ability to capture the nonlinear properties of bones. Future work will incorporate deformation data from CT images to better estimate these properties, improving model accuracy and applicability. Including more diverse patient data, such as different ages and sexes, will enhance the robustness and generalizability of the model. Future studies will also explore the integration of additional loading conditions and the role of ligaments and cartilage endplates in the biomechanical behavior of the spine. This will provide a more comprehensive understanding of spinal mechanics and further validate the model's applicability to various clinical scenarios. Expanding this method to other regions of the spine and different patient populations will also be a focus of future research, ensuring broader applicability and impact. Future work will focus on incorporating phantom-based calibration to enhance the accuracy of CT-derived material properties. This approach will allow for improved estimation of bone density and mechanical properties, enabling more personalized finite element models and reducing variability associated with literature-based assumptions.

## Conclusion

This study demonstrates the integration of Finite Element Analysis (FEA) and Physics-Informed Neural Networks (PINNs) to predict the material properties and biomechanical behaviors of the human lumbar spine. This approach achieved high accuracy (94.30%) and eliminated the need for extensive manual work in lumbar spine FEA modeling. The proposed automated coding approach not only simplifies the process by automatically segmenting and meshing the lumbar spine, setting different contacts and incorporating endplates and discs, but also maintains high accuracy, making it a more efficient method for biomechanical modeling. By utilizing patient-specific CT data, it provides precise material properties, segmentation, stress fields, and surgical plans without invasive procedures, significantly benefiting clinical practice.

However, the current model does not use deformation data from CT images, limiting the ability to capture nonlinear bone properties. Future work will incorporate this data to improve accuracy and applicability. Additionally, expanding the model to include diverse patient data and additional loading conditions will enhance its robustness and generalizability, further validating its clinical relevance.

## Declaration of competing interest

The authors declare that they have no known competing financial interests or personal relationships that could have appeared to influence the work reported in this paper.
